# The Microbiota–Gut–Brain Axis Heart Shunt Part I: The French Paradox, Heart Disease and the Microbiota

**DOI:** 10.3390/microorganisms8040490

**Published:** 2020-03-30

**Authors:** Mark Obrenovich, Bushra Siddiqui, Benjamin McCloskey, V. Prakash Reddy

**Affiliations:** 1 Research Service, Louis Stokes Cleveland, Department of Veteran’s Affairs Medical Center, Cleveland, OH 44106, USA; 2 Department of Chemistry, Case Western Reserve University, Cleveland, OH 44115, USA; 3 The Gilgamesh Foundation for Medical Science, Research and Education Cleveland, OH 44116, USA; ben@engineindustries.com; 4 Department of Medicinal and Biological Chemistry, College of Pharmacy and Pharmaceutical Sciences, University of Toledo, Toledo, OH 43606, USA; 5 Departments of Chemistry and Biological and Environmental Sciences, Cleveland State University, Cleveland, OH 44115, USA; 6 North East Ohio College of Medicine, Rootstown, OH 44226, USA; bsiddiqui@neomed.edu; 7 Department of Chemistry, Missouri University of Science and Technology, Rolla, MO 65409, USA; preddy@mst.edu

**Keywords:** French paradox, polyphenol, cerebrovascular, heart brain shunt, microbiota-gut-brain axis, Trimethyl-Amine-N-Oxide, TMANO, TMAO, co-metabolism, Alzheimer, vascular dementia, redox, HMG-Co A, red wine, blood brain barrier, Celiac disease, leaky gut

## Abstract

It has been well established that a vegetarian and polyphenol-rich diet, including fruits, vegetables, teas, juices, wine, indigestible fiber and whole grains, provide health-promoting phytochemicals and phytonutrients that are beneficial for the heart and brain. What is not well-characterized is the affect these foods have when co-metabolized within our dynamic gut and its colonizing flora. The concept of a heart shunt within the microbiota-gut-brain axis underscores the close association between brain and heart health and the so-called “French paradox” offers clues for understanding neurodegenerative and cerebrovascular diseases. Moreover, oxidation-redox reactions and redox properties of so-called brain and heart-protective foods are underappreciated as to their enhanced or deleterious mechanisms of action. Focusing on prodromal stages, and common mechanisms underlying heart, cerebrovascular and neurodegenerative diseases, we may unmask and understanding the means to better treat these related diseases.

## 1. Introduction

Part I, of a two-part series of concept papers, focuses on novel findings involving nutrition, biochemistry, microbiology and metabolism to suggest a heart shunt, as occurs when two organ systems are coupled and in this case within the microbiota-gut-brain axis. Here, we explore the bacterial species that contribute to disease and to health and suggest oxidative and inflammatory mechanism drive heart and brain disease and that wine polyphenols help protect us from bacterial-derived deleterious metabolites. Exploring aspects of co-metabolism within the microbiota-gut-brain axis and metabolites from prosaic foods could lead to a unifying hypothesis for age-related diseases and advance our understanding of vascular dementia, neurodegeneration and heart disease. 

When one considers ways to affect and modulate one’s own health, one must start with diet and lifestyle. After this, we then turn to the host resident microbial ecology. Findings from host digestion with gut microbiota and mycobiota co-metabolism suggest we can explore mechanisms of disease and new treatment approaches to modulate health. To simplify our understanding of the two most common and extremely complex diseases in the world today, namely coronary heart disease (CHD) and Alzheimer disease (AD), we describe a shunt between the heart and the brain, where commensal microorganisms though the microbiota-gut-brain (MGB) axis do affect both the heart and the brain, which are coupled through the vasculature. 

The gut microbiota has largely coevolved, in beneficial ways with their hosts, where they co-metabolize dietary foods in symbiosis. The relationship provides both a niche for the microbes, and extracts vitamins, vital nutrients and energy from diverse dietary sources for their hosts and themselves. In fact, it could well be that some microbe-provided nutrients have negated the need for *de novo* synthesis by their hosts and the symbiont’s production of some metabolites demonstrate antagonistic pleiotropy [1], which are just vestigial remnants of an evolutionary past. The case could certainly be argued for some vitamins and cofactors and bacteria may even have contributed to loss of functional genes for some of these nutrients during evolution. Regardless, it is without question that animals, plants and bacteria coevolved over time to survive, adapt and reproduce. It is this intrinsic co-metabolism that we feel hold the answers to our unrelenting questions of how and why we are plagued with diseases in the first place and what can be done to affect change in neurodegenerative pathobiology and its prevention. 

We know the microbiota are involved in the early development of the blood-brain barrier [2]. In fact, homeostasis and establishment of the circulatory system and immune system are influenced by gut bacteria and their metabolites [3]. Further, oxidative and inflammatory stressors are key propagators of disease pathogenesis for the brain and the heart. Therefore, antioxidant foods and anti-inflammatory agents are expected to provide the most impact on disease prevention and treatment and much of this is expected to be derived from food. Conversely, a poor diet could do just the opposite, contributing to disease by similar mechanisms. 

## 2. The French Paradox

In search of beneficial dietary sources for health-promoting compounds led to fruits, vegetables, wine and grape juice, as their consumption was inversely correlated with coronary heart disease (CHD). Moreover, within populations who consumed a lot of red wine, it was observed that a paradox of sorts existed within one population in particular because they consumed foods known to be deleterious for heart health, yet had little heart disease. Thus, the cardio-protective effects of wine began to be enthusiastically studied and has come to be known as the “French paradox [4]. This peculiarity largely surrounds the observation that the French diet is rich in lipids and fatty foods but French people have relatively low morbidity and mortality from cardiovascular and other heart-related disease. How the French are able to consume very rich fatty and saturated lipid-rich foods in greater quantities than most other groups, while remaining relatively unaffected in their cardiovascular health remains a mystery and at the same time their Alzheimer disease risk is lower than some other populations and certainly lower than those that consume a very high fat diet [5]. Nevertheless, we know that what is good for the heart is also good for the brain, most likely involving the shared cerebrovascular system and this is a more important consideration today, since, world-wide, one in three deaths is attributed to cardiovascular disease [6].

As the popularity of the French paradox increased, so did the need for evidence-based research. It is believed the French paradox is explained by the red wine constituents, which protect the French from heart attacks and have other health benefit. These foods are rich dietary sources of a class of compounds called flavonoids, which are small molecular weight polyphenols. To date, no less than 8000 phenolic structures having been identified in plants [7]. Polyphenols are classified by the number of phenol rings and the chemical substituents attached to these rings. Suggested mechanisms of action for polyphenol constituents are related to their antioxidant capacity, anti-inflammatory activity and signal transduction pathway activity [7]. Some of these compounds are reported to have low dose hormetic effects activating stress response pathways when exposed to reactive oxygen species, free radicals, superoxide anion, hydrogen peroxide, reactive nitrogen species, nitric oxide, peroxynitrite anion (ONOO) or hydroxyl and peroxyl radicals [8]. Other sources of ROS include xanthine and flavin oxidases and cytochrome P450, which subsequently activate ROS elimination systems and at the same time [9] Fenton reactions can be produced during food digestion [9]. However, finding the hormetic zone within microbial co-metabolism or with bacterial toxin exposure is likely far off from the scientific community’s radar. Nevertheless, how the same foods impart health and play a part in disease is not a mutually exclusive concept and our review will demonstrate that the gut microbiota and oxidative stress-mediated inflammatory process is likely the deciding regulator within these apparently disparate findings.

## 3. Gut Redox Pairs and the French Paradox

The topic of wine is frequently mentioned in nightly news reports. Often broadcasters cite the latest study to recommend the consumption of a glass of wine per day, or beer for that matter, will benefit human brain and heart health. Nevertheless, studies often have many limitations, but even fewer mention metabolism involving bacterial action or suggest redox properties of the constituents or metabolites of those prosaic foods. Redox potential, as in redox-paired compound activity, is an important environmental feature. The ability for redox pairs to find each other and have any activity is readily affected by host and bacterial metabolism and the microenvironment where the interaction occurs. When considering redox pairs for wine constituents, their potential when paired with deleterious reactive species is an important but under-appreciated feature of the gut environment. Both host and microbial enzymatic and non-enzymatic action, coupled with varying degrees and states of oxygen tension, are but a part of the dynamic involved in understanding protective mechanisms for polyphenols. Importantly, dietary polyphenols that are not taken up by bacteria for metabolism, pass through the small to the large intestine the gut lumen and are taken up by enterocytes or other intestinal cells enter the gut lumen and are subject to host and liver metabolism and modification, such as phase 1 and 2 conjugation.

These phenolic compounds certainly become oxidized themselves to quinones and protect from oxidation-mediated damage but, quinones are potentially oxidizing as well. It would be a paucity of antioxidants or other mechanisms that would either contribute to or protect against disease pathogenesis. Few have considered whether microbial-host metabolism, or redox properties of bacterial-derived molecules, would interact with prosaic foods to either antagonize or synergize in disease pathogenesis. We suggest some of the action occurs either by directly possessing enzymatic or regulatory substrate interaction or by forming complexes, such as chelating redox metal-bound moieties, acting as glycation intermediates or via other oxidation-quenching mechanisms. Since polyphenols are powerful antioxidants and scavengers of ROS and RNS they protect extracellular lipids, proteins and nucleic acids from redox damage but questions remain as to whether some antioxidants or phytochemicals potentially could do more harm than good, as increases in glycation-mediated protein damage have been reported [8]. Far less is known about whether or how phenolic acids and phytochemicals pass barriers to act intracellularly or in brain compartments. In aerobic cellular systems, the redox potential and redox pairing metabolites are largely governed by extracellular reactive oxygen and nitrogen species, whereas in anaerobic systems, the bacterial species and supply of their reducing metabolites reflect the net antimicrobial potential of gut contents [10]. Generally, carbon dioxide, sulphur dioxide, zinc oxide and oxygen elevate redox potential and hydrogen depresses it. Therefore, it is wise to consider these redox pairs and interactions with foods and their metabolites when thinking of mechanisms of action. We suggest further study and the development of next generation mass-spectral approaches and metabolic interactome methodologies be devised.

Since we cannot escape oxidative metabolism and oxidation is a common biochemical enzymatic mechanism, we must consider oxidation-reductions *in vivo*. Oxidation reduction or redox potential (Eh) is measured in millivolts and represents the affinity of electron transfer to or from a chemical species in solution. The ability to either gain or lose electrons in that particular environment is concentration and temperature dependent. Chemical species with higher, more positive, reduction potentials than a particular species introduced into the system trend to gain electrons from the new species, become themselves reduced by the oxidizing species and solutions with lower, more negative, reduction potential tend to lose electrons to the new species and become themselves oxidized by reducing the new species [11]. 

Within the gut, intestinal segments, the crypts lining it and other environmental niches within the gut continuum have changing redox states and differences in oxygen tension and the resident bacteria play a large role in maintain redox potential within these environmental segments. Moreover, it has been shown that imbalances between pro-oxidative and anti-oxidative mechanisms play a key role in the pathogenesis of irritable bowel disease and related intestinal damage [12,13]. For instance, strict aerobic bacteria are largely active at positive redox values, whereas strict anaerobes are generally active at negative Eh values and facultative anaerobes can be active at both positive at negative Eh values and in the presence of oxygen bearing nitrogenous or sulfurous inorganic compounds. In cellular metabolism oxygen, nitrate, chlorite, nitrite, iron, copper, sulfate, hydrogen peroxide, manganese and CO_2_ serve as inorganic oxidants, while reduced inorganic compounds, metals and organic substrates like hydrogen sulfide (H_2_S) serves as reductants [12] Probiotic bacteria do scavenge ROS and degrade Fenton and Haber-Weiss reaction products such as superoxide anion and hydrogen peroxide, which are not restricted to their metal chelating ability and significantly attenuate exopolysaccharide (EPS)-produced oxidative damage in a rat model of colitis [14].

Other central nervous system (CNS) antioxidants include diffusible bioactive gasses like molecular hydrogen (H_2_) as dihydrogen and H_2_S, which have having antioxidant activity, such as reducing hydroxyl radicals and peroxynitrite from microbiota potentially generate up to 1 L per day during some fermentation processes [15]. Small amounts of H_2_S are produced by host metabolism, but it is mostly by hydrogen-producing bacteria, which include anaerobic cocci, certain strains of *Clostridium* and some *Enterobacteriaceae*, among others. H_2_S can inhibit Complex IV within the mitochondrial electron transport chain [16,17]. Production of H_2_S is usually accompanied by symbiotic co-metabolizing species that consume H_2_S as in the case of sulfur and sulfate-reducing bacteria, methanogens, and acetogens bacteria [18]. The production of H_2_ has intra- and inter-individual variability in relation to microbial composition and dietary choice. Since H_2_ is by far gut bacteria-derived and not of human origin [16] it is plausible that gut dysbiosis results in decreased protective H_2_ production for the CNS, vulnerable neurons and increases oxidative stress susceptibility in all ROS-related disorders. This is supported by Parkinson disease findings [19]. Some oxidizing enzymes, such as dioxygenase, polyphenol oxidase (PPO) and quercetinase, mediate catabolic conversion of select polyphenols in the anaerobic colon. Since the colon is an anoxic environment, as compared to the small intestine, these enzymes depend on molecular oxygen for their catalytic activity yet activity occurs here. Perhaps there is some slightly higher oxygen tension in the gut wall or crypts. Nevertheless, stochastic and oxidative mechanism occur under conditions of very low oxygen tension in the gut [20].

Redox potential is an important environmental feature, affected by both host and microbial actions, and redox reactions are important for determining carbon and nutrient cycling in bacterial and other systems [21]. In solution, redox conditions affect the nutrient and free metal ion solubility and availability. For instance, gut bacteria are known to interfere with the intracellular NADH/NAD+ ratio in their competitors, such as *Clostridium acetobutylicum* [22]. They accomplish this by using different substrates reducing capacity, e.g., pyruvate, glucose and glycerol [23]. Because molecular oxygen, involved in keeping metabolism in flux, synthesizing unsaturated fatty acids/sterols and for maintaining cell membrane integrity and function, regulating the levels of dissolved oxygen in solution is important for host and bacterial metabolism. This holds for microorganisms that propagate under optimum physiological redox conditions in the gut. Moreover, antibiotics change host and microbial functioning and can perturb gut redox potential, which increased within hours after dosing in mice [10] and this group found shifts in redox potentials were specifically attributable to bacterial suppression and blooms of the bacterial family *Enterobacteriaceae* species in a host-free human gut model of the microbiota. 

Molecular oxygen (O_2_) is considered to be a driver of gut dysbiosis [24] and we know gut microbes contribute to redox signaling [25]. The gut microbiota oxidation-reduction potential, e.g., capacity for the microbiota to gain electrons, also influences the homeostasis of the intestinal-blood barrier [10], while at the level of the brain and CNS, modulates intestinal ROS levels via the vagal, cholinergic and anti-inflammatory pathways [26,27]. Further, oxidative and inflammatory stress can lead to distal breaches for other immune privileged barriers in humans [3].

Since there is a direct link between fecal redox potential, human digestive microbiome and its health, Million and co-workers explored links between anaerobic depletion, increased redox potential in various systems and during severe acute malnutrition [28]. They found intestinal redox-related indicators were significantly correlated with the distribution of gut microbiota and diet is a key consideration. Interestingly, processed meats are considered non-healthy because many contain heme-iron, an oxidant and source of redox active metal, and high fat, salt and nitrite content. In particular, red meat with abundant nitrates form free radicals in the human gut. Researchers tested various natural compounds, such as tea polyphenols and *Polygonum cuspidatum* and rosemary extract to counteract the effects oxidation in the gut and experiments were carried out to add polyphenolic compounds into meat processing to improve upon its relative unhealthiness [29]. This group found the addition of polyphenols and extracts reduced the oxidation-reduction potential of meat products and increased their antioxidant status. 

Moreover, red wine, red grape juice and prune juice also affects gut oxidative stress as they are known to have profound inhibitory effects on iron bioavailability [30]. These inhibitory effects are likely due to high levels of particular polyphenols that bind redox-active iron species and prevent its absorption. Many things feed bacterial growth and during sepsis or anthrax exposure, free iron feed bacterial growth and these processes are life threatening. Our acute phase reactive proteins, like C-reactive protein and those that bind ferritin and other iron binding proteins are upregulated to sequester microbial growth-enhancing elements like iron [31]. This is clearly an evolutionary defensive approach in managing infections and sepsis in animals. Together, these findings show a potential to manipulate the gut microbiota through managing bacterial respiration and polyphenol administration. 

## 4. Possible Mechanisms for the French Paradox

When considering potential mechanisms for the French paradox, it is important to note that wine is a natural statin. The effects of red wine and both of the aforementioned statins are directly traced back to their ability to inhibit 3-hydroxy-3-methylglutaryl coenzyme A reductase. We call this so-called HMG-Co A reductase inhibitor action. HMG-Co A is an NADPH-dependent enzyme and the rate-limiting step of the metabolic pathway that produces cholesterol and other isoprenoids, namely, the mevalonate pathway. HMG-Co A reductase reduces cholesterol and biosynthesis of ubiquinone and other molecules in this lipid synthesis pathway like isoprenoid production as well as the production of many other biomolecules. Isoprenoids activate a Rho family member of G-proteins involved in inflammation and these compounds then lower inflammation. The author has shown a clear connection between G-protein receptor kinase 2 (GRK2) and AD [32], which was the basis for the concept of the heart-brain connection in vascular AD, vascular dementia (VaD) and other forms of Alzheimer disease [33]. 

The French paradox and polyphenol involvement could be further explained specifically by vitisin A and vitisin B, which have strong inhibitory activity against HMG-Co A reductase [34]. Moreover, *Vitis vinifera* has inhibitory effects on HMG-Co A reductase through metabolites of the mevalonate pathway [8], which are unique from its statin-like activity and those affecting cholesterol levels, vascular tone hemodynamics or potential control of vascular function [35]. When Roullet and colleagues explored Lovastatin in rat heart explant tissue, they found the opposite effect in vivo and in vitro as Lovastatin increased blood pressure, enhanced vascular response to norepinephrine, and impaired endothelium-dependent and independent relaxations. On a whole, they found mevalonic acid metabolites decrease blood pressure in the whole animal without significant change in plasma cholesterol [35]. Thus, pharmacological inhibition of mevalonate production as a means to lower plasma cholesterol may have an adverse impact on cardiovascular risk factors, such as blood pressure. Moreover, the use of polyphenols and wine to block cholesterol production should be weighed carefully in terms of cost benefit.

Alcohol maybe protective for CHD by increasing serum high density lipoprotein, decreasing cholesterol levels and by inhibiting platelet reactivity [36]. Aside from alcohol in the wine, we described many wine constituents that may explain the French paradox [37], such as polyphenols, which do explain some of the health disparity of the so-called paradox as they have particular importance to diabetes, cancer, aging and neurodegenerative diseases. This may be due, in part, through multifactorial effects on post-translational modifying enzymes that affect histone protein acetylation patterns for example [38]. However, these same health-promoting compounds are attributed to the so-called Mediterranean diet as well, which is one generally rich in vitamins, high-fiber, fruit and vegetables [39,40]. 

Many fruits and juices contain phenolic acids, polyphenols and indigestible carbohydrates, such as pectin, hemicellulose and various polysaccharides. It is this aspect of the diet that both supports select gut microbiota species and colonization through co-metabolism, whereby the gut microbiota can impart many beneficial effects on health through sustained co-metabolism of health-promoting foods. The microbial fermentation by-products are short chain fatty acids (SCFAs), particularly acetate, propionate, and butyrate [41]. These prebiotics, in fact, may act synergistically to modify colonic and intestinal microbiota, which form a symbiosis and benefit human health [42]. In that regard, we found protective aspects of butyrate from the prebiotic potato starch with *Faecalibacterium prausnitzii* on *Clostridium difficile* infection and damage [43]. Others found prebiotic effects, using 16S rRNA pyrosequencing of the microbial DNA prepared from stool co-incubated with select fruits when fermented with feces from 10 donors [42]. This group found an immediate and long-lasting reduction in the abundance of all members of the Proteobacteria phyla as well as some members of the Firmicutes phyla and Bacteroidetes phyla, which were originally present in the original fecal inoculum. Further, the abundance of *Ruminococcaceae* or *Firmicutes* decreased in 24 h by half. In contrast, *Bacteroidaceae*, *Lachnospiraceae* and *Veillonella* members of the Firmicutes phyla and *Coriobacteriaceae* of the *Actinobacteria* phyla increased over 24 h. The *Lachnospiraceae* levels were sustained for up to 48 h and *Coriobacteriaceae* and *Bifidobacteraceae Actinobacteria* increased [42]. Further, they showed that *Proteobacteria, Actinobacteria, Firmicutes* and *Bacteroidetes* all compete for dietary carbohydrate in a closed system. *Proteobacteria* have a preference for host carbohydrates and mucin-derived and secondary metabolites.

When fermented kiwifruit was added, SCFAs increase, while lactate and after 24 h succinate concentrations declined and most of the genera observed produced acetic acid [42]. Propionic acid is a SCFA product of *Bacteroidetes* fermentation and from *Veillonella*, members of the phyla *Firmicutes*. Butyrate, produced mainly by the Firmicutes subset, among others, features members of the *Lachnospiraceae* family [42]. Additionally, in the aforementioned study by Juliet and colleagues, by-products of fermentation contributed to the first step in microbial colonization by modulating both microbial numbers and gut flora ecology through adhesion of different bacteria to the gut wall [44]. In the gut, this offers stable colonization and these adhesion effects are consistent with the work on pectin, whereby fractions rich in galactose, arabinose, and galacturonic acid enhanced the adhesion of *Lactobacillus rhamnosus* to Caco-2 cells in vitro, but inhibited the adhesion of *Salmonella enterica* [45] The differential adhesion of pathogens vs. commensal establishment is another example of how our niche microbiota in well matched to us and contribute to our health.

## 5. Redox Active Polyphenols and Phenolic Acids

**Figure 1:**
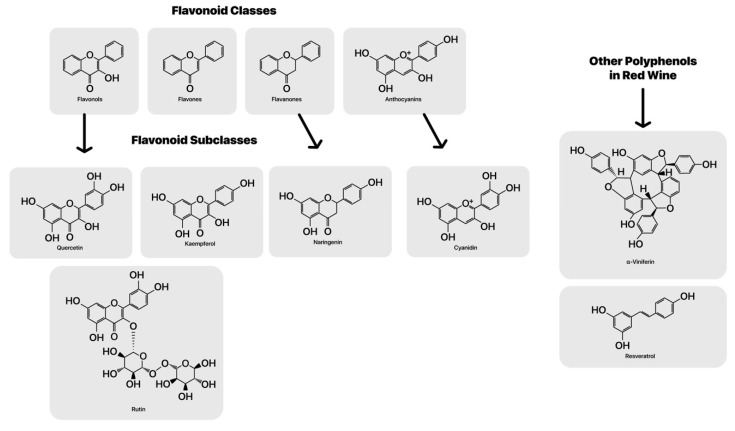
The major polyphenolic compounds in red wine, including flavonoids, flavones, flavanols and anthocyanidins.

Oxidants are chemical species able to remove electrons from electronegative atoms or other molecule’s electrons and then accept those electrons the scavenged species then become oxidized [46]. But there is a double-edged sword to many redox active compounds in the gut and some may even to contribute to heart disease through the redox action of select microbiota. In red wine, the main polyphenolic constituents are a class of phytochemicals called flavonoids or flavones and flavanols, anthocyanidins [47], which includes their oxidation products [48] (See Figure 1). These small molecular weight flavonoid class of compounds provide antioxidant activity based on redox potential. They serve as electron donors to select free radical derived oxidants, such as hydroxyl radicals and peroxyl radicals due to favorable reduction potentials of peroxyl radicals [49]. Jovanovic and colleagues used a model in aqueous solutions with azide radical induced single electron oxidation to generate phenoxyl and flavonoid radicals and investigate their acid–base and redox properties. This may also help protect the French from lipid peroxidation and heart disease [50]. Moreover, xenobiotic molecules could affect the CNS and brain at both the blood-brain-barrier interface [51] and from gut dysbiosis, which could be a cause and a consequence of increased levels of oxidative stress since anaerobes thrive in the presence of electron acceptors [52].

A reaction that is well-quenched by many polyphenols is the Fenton reaction, which involves hydroxyl radicals. This and other oxidative mechanisms affect redox signaling, activate survival pathways mediated by “redox sensors” and modulate the expression of certain anti-oxidant enzymes, which are kept under control by soluble and insoluble redox sinks, i.e., compounds able to quench oxidative stress or free radical species. The endothelium-derived Nitric oxide (NO), is a relaxing factor and free radical and a neurotransmitter of the non-adrenergic non-cholinergic enteric nervous system is the of the autonomous nervous system [53]. The systems modulate the level of oxidative stress within the intestine via the vagal cholinergic anti-inflammatory pathway [54]. Importantly, the central and enteric nervous systems interact with MGB axis via gut microbiota to regulate neurotransmitter and hormone metabolism and the vagus nerve is a key node. The resulting communication signaling contributes bidirectional control of gastrointestinal tract functions and related brain behavior. In the opposing direction, communication affects afferent vagal nerves, which express many receptors for gut peptides such as ghrelin serotonin and leptin to name a few. The enteroendocrine cells that secrete peptides and small molecules are regulated to some degree by the microbiota and the MGB axis.

An important, but less concentrated constituent of red wine, is the phytoalexin resveratrol, which is a naturally occurring phenolic acid and effective lipid-soluble antioxidant. In the recent past, interest in resveratrol grew due not to its antioxidant properties but due to is activity as a histone deacetylase, which was expected to be a new AD therapy [55]. Although it is not the most abundant polyphenol in wine, trans-resveratrol has been reported to prevent Angiotensin II-induced hypertension and endothelial dysfunction. It has also been shown to prevention of vascular nicotinamide adenine dinucleotide phosphate (NADPH) oxidase induction [56]. Moreover, Vigili and co-workers were the first to demonstrate neuroprotection in rodents with trans-resveratrol form kainic acid injections in the hippocampus and olfactory cortex [57].

However, we suggest alternative mechanisms may be favored and understanding examples from plants and fruits may provide insight. Since flavonoids are ubiquitously synthesized in all photosynthetic plants as an acute response phase compound under various stressors and during environmental attack. Similarly, these same compounds, when ingested, are likely to protect against the same environmental assaults and stressors common in human disease pathogenesis. One mechanism helpful to plants and humans likely involves the ability for flavonoids to sequester and chelate redox-active metals in response to heavy metal stressors [58]. Moreover, they are also produced during ionizing radiation and during microorganism infection, where they have clear activity against pathogens like bacteria, fungi and viruses [59]. However, the overall bioavailability of flavonoids, when taken orally, may be lower than preferred.

Other putative factors in red wine that may be important to prevention cardiovascular disease and risk are folic acid and nitric oxide, which some suggest are involved in the French paradox [60]. In bovine aortic endothelial cells, vascular homeostatic changes occur when ET-1 is over-expressed and leads to changes in distribution and in tyrosine phosphorylation [61] which may implicate red wine components in multiple signal transduction pathway action or inhibition. In that regard, cellular targets of flavonoids are likely kinases involved in signal transduction pathways like MEK, Raf and PI3K [61]. Recently, interest in microbial metabolism of polyphenols, phenolic acids and similar red wine components has grown and is a promising area of research for medicinal chemistry [62].

Important in the red wine debate is its ethanol content as alcohol participates in free radical reactions within biological systems though production of alkoxy-free and hydroxyl-free-radicals as the initial reaction products [63]. However, ethanol may enhance cellular oxidative stress and be deleterious under some conditions by producing superoxide anion and hydrogen peroxide. Conversely, moderate ethanol consumption is considered beneficial, because it is known to decrease inflammation and inflammatory markers in adipocyte tissue explants and attenuated whole-body inflammation and lowered soluble tumor necrosis factor receptors in vivo [64] and does lower other inflammatory mediators, such as soluble tumor necrosis factor (TNF) receptors l and ll and other cytokines [65]. However, polyphenols are not the only protective compounds in a vegetable-rich diet. Multiple antioxidant properties of flavonoids have been noted each with different mechanisms of action. In particular, they trap reactive oxygen species and inhibit enzymes involved in the production of oxidative stress. Flavonoids also block radical formation that occur through Fenton type reactions and even regenerate other antioxidants, such as α and γ-tocopherols (vitamin E). Radical trapping experiments by Drouza and colleagues, demonstrated that the vanadium complexes in edible oil activate the one electron reduction of dioxygen to super-peroxide radical anion that reacts with lipids to form lipid hydroperoxides and react with the phenols contained in the oils [66]. ROS-trapping mechanisms, through the oxidation of flavonoids to short-lived radicals, regenerates vitamin E. These radicals usually decay quickly into non-radical oxidation-degradation products via complex pathways and vitamins E and C both are labile, acting as antioxidant as well as pro-oxidants. 

## 6. Lipid Metabolism, Oxidation and the French Paradox 

Human plasma contains many sulphur-containing proteins with intrinsic antioxidant properties, including glutathione peroxidase, thiols, some in their active site, as well as transition metal binding proteins, albumin and many oxidases and reductases that protect endogenous lipids proteins and nucleic acids from damage [67]. Inhibitors of lipid peroxidation and oxidation damage to low-density lipoprotein (LDL) are useful phyto-protective compounds and polyphenols, particularly the catechins, are effective chain-reaction breaking antioxidants and scavengers of free radicals and polyphenols do this as well. The mechanism is to spare LDLs, which are rich in polyunsaturated fatty acids (PUFA)s and are a major cholesterol carrying lipoprotein in plasma. Elevated levels of LDL, PUFAs and cholesterol directly contribute to atherosclerotic disease and lipid oxidation is strongly implicated in this process. The oxidation process promotes the accumulation of cholesterol-esters on arterial walls and in endothelial cells, which is the first step in the LDL oxidation cascade which includes the oxidation and degradation to aldehydes of polyunsaturated fatty acids. Here, monocytes catalyze the process becoming resident macrophages where they directly contribute to bystander generation of reactive oxygen species damaging the vascular microenvironment as they attempt to remove the plaque deposits through immune clearance mechanisms. The activation of several phox-regulatory complex of enzymes, and oxidative bursts from macrophages, feed this cascade forward. When macrophages ingest oxidized deposits, such as oxidized LDL and oxidized apolipoprotein small a, they transform into so-called foam cells, which we see as fatty steaks in affected atherosclerotic vessels and polyphenols likely could prevent this cascade in the first place [67]. 

Lipid peroxidation and oxidative stress with platelet aggregation and LDL oxidation, has been inhibited by lipid soluble antioxidants, such as resveratrol and wine [67]. Further, inhibition of LDL oxidation by flavonoids is suggested by the localization of catechins near the membrane surface and by scavenging of aqueous radicals since they are ideally located in the aqueous phase interface where they prevent the oxidation of the tocopherols leaving tocopherols to function in cells as a lipid peroxyl radical scavenger [68]. Resveratrol is capable of scavenging lipid hydroperoxyl free radicals, hydroxyl and superoxide anion radicals [69]. Others show in vitro that amelioration of oxidative damage induced by hydrobutoxide was greater with resveratrol than with common redox-labile antioxidants vitamin C and vitamin E [70]. Many enzymatic and non-enzymatic antioxidant defenses protect circulating LDL and lipoproteins from oxidation, which is enhanced and catalyzed by circulating free transition metals through Fenton and Haber-Weiss reactions as metallic ions remain redox active even when protein bound [71]. Oxidized LDL can be reduced by antioxidants with minimal lipid peroxidation. Inhibitory properties of polyphenols toward LDL oxidation via Fenton chemistry can be calculated by several oxidation-potential indices and followed biochemically. In Fenton reactions, this is divided into three phases; lag, propagation and decomposition, taking into account the initial number of conjugated dienes, lag time, maximal rate of oxidation, maximal number of dienes formed, and so on [8].

## 7. Alcohol and Red Wine Polyphenols Affect the Microbiota

The French have perhaps the highest intake of total alcohol and wine for any developed country [39]. Polyphenols are a particularly interesting general class of plant compounds that contain multiple phenol groups in their chemical structure. Certain antioxidant characteristics found in red wine were attributed to key phytochemicals that offer beneficial effects for modulating human health. Two main types of polyphenols are known, namely regular flavanols and anthocyanidins. Stilbenes, like resveratrol, are known but they have properties other than as antioxidants. When we study gut microorganisms, often through DNA sequencing to reveal which species are present in a particular animal or system, we also explore how the microbiota, mycobiota and other microorganisms respond individually or collectively to a particular environment or milieu as they co-metabolize nutrients with their hosts. We combine these approaches with analytical methods to focus on the small molecules produced as microbes metabolize food and host metabolic products. Beyond genomics and computational biology, we also use mass spectroscopy and bioinformatics to explore biochemically and physiologically the production and movement of these molecules to the brain or other host compartments. These small molecules are biomarkers of metabolism, where they could serve to chart health, disease, treatment efficacy or the progression of syndromes or sequelae related to co-metabolism between a host and its resident microorganisms. We call this aspect of analytical science metabolomics or the systems biology of metabolism [62].

Several classes of polyphenols are abundant in many foods, including tea, coffee, fruit, vegetables and chocolate [72,73]. Those opposed to alcohol likely question why adding polyphenols to our diet in the form of red wine, versus taking non-alcoholic sources or purified phenolic compounds in pill form, would be at all beneficial or different. One important finding came after the work of Guarente and Sinclaire, who isolated resveratrol and packaged it as a drug and fountain of youth dietary supplement. Resveratrol’s drug application presents challenges for the pharmaceutical industry due to its poor bioavailability, poor solubility and adverse effects. In that regard, dietary resveratrol in food is present in more stable and bioavailable glycosylated forms, which prevent its enzymatic oxidation in the gut. Furthermore, intestinal cells only absorb the resveratrol aglycone, which makes it more effective as a whole food, at this time, rather than as an isolated compound [74]. 

More importantly, it was the Le Roy study that revealed grapes compared to other foods have a much richer concentration of polyphenols in their skin, specifically in red grapes versus white grapes and red wine had a higher polyphenol content compared to non-alcoholic grape juice [75]. Results of wine’s effect on the microbiota show that red wine consumption significantly increases the concentration of Firmicutes, Bacteroidetes, and Proteobacteria phyla but not in those from the gin or alcohol-depleted red wine group [76].This finding demonstrated the significance that small ethanol doses with polyphenol intake is likely needed to create some of the observed changes in the gut microbiota [76]. 

When considering the predominant red wine polyphenols studied, including the flavan-3-ol monomers and proanthocyanidins, we often find associated antimicrobial activity, which may be another reason why wine may be protective against heart disease as they affect the microbiota by exerting inhibitory effects through binding to certain bacterial membranes [77,78]. As some of these compounds reach high levels in the blood periphery and select organs, biologically significant concentrations do affect the host and the microbiota. It is important to note that wine and tea contain larger molecular weight tannins, phenolic concatemers and polymeric polyphenols. Condensed and hydrolysable tannins, the proanthocyanidins, are known to impair nutrition, bind proteins with soluble or precipitated complexes and affect intestinal absorption of foods including lipids [79]. The non-absorbable polyphenols, non-hydrolysable and hydrolysable tannins and dietary polyphenols have provoked new ideas as to their physiological function, interaction with lipids and metabolism involving the microbiota-gut [80]. We know a high fat diet induces metabolic disorders, but it also induces gut microbial dysbiosis in mice, however, some polyphenols from tea possess the ability to regulate dyslipidemia and gut microbiota dysbiosis [81]. 

Polyphenols, such as those in tea, the theaflavins, are considered to have antimicrobial activity and underlie intestinal mechanisms that significantly ameliorated hyperlipidemia, improved the expression levels of hepatic lipid metabolism genes and modulate the gut microbiota in mice [81]. The exact mechanisms for ameliorating hyperlipidemia are largely unknown, but potential health benefits surrounding natural compounds are very much dependent upon absorption and disposition to target tissues and cells. Thus, we suggest a caloric restriction mimetic effect, of tannins and resveratrol, might be based simply on their ability to limit intestinal absorption of food [82]. One other underlying mechanism rely on tea polyphenol maintenance of the intestinal redox state. Biomarkers for intestinal redox state include an unidentified genus of *Lachnospiraceae, Bacteroides*, *Alistipes*, and *Faecalibaculum* and suggest novel insights into the mechanism of tea polyphenols on intestinal redox homeostasis [83]. 

Studies show that regular moderate red wine consumption significantly modulates the growth of select gut microbiota in humans by inhibiting non beneficial bacteria from the human microbiota and potentiating the growth of probiotic bacteria. In a randomized, crossover and controlled intervention study, researchers evaluated the effect of moderate intake of red wine polyphenols on groups of human gut microbes [76]. Results of this study revealed that after daily red wine polyphenol consumption over 4 weeks, there was a significant increase in the number of Blautia, *Coccoides-Eubacterium rectale*, *Bifidobacterium, Eggerthella lenta, Bacteroides uniformis, Enterococcus, Bacteroides*, and *Prevotella* groups and importantly a decrease in the *Clostridium* genera. These include *Clostridium histolyticum,* which produces collagenase that degrades animal tissue; *Clostridium difficile*, which causes severe diarrhea and death; *Clostridium perfreingens*, which causes gas gangrene; and other species responsible for disease in humans [76]. *Clostridium perfringens* is closely related with progression of colonic cancer and the onset of inflammatory bowel disease [83], which is decreased after red wine consumption. *Eggerthella lenta* and *Bacteroides uniformis* is associated with antiproliferative effects on human prostate cancer cells and proliferation was decreased after red wine consumption [84]. *Blautia, Coccides-Eubacterium rectale* species was found important for the prevention of colon cancer [85] or ulcerative colitis [86] due to the presence of butyrate [87], which increased after red wine consumption. In relation to bacterial composition, resveratrol another red wine component that was shown to have beneficial effects on metabolic syndrome-related alterations in composition of bacterial species. Further, this wine compound is generally associated with beneficial metabolic outcomes [88] and improvements in gut microbial composition generally [75]. Results from twin studies revealed that the twin that consumed more red wine had a healthier gut microbial pattern and a lower risk of obesity and bad cholesterol compared to their twin counterpart who consumed less red wine. Another study exploring the effects of red wine in the microbiota gut of nearly 3,000 people comprising of three different countries namely, United Kingdom, USA, Belgium, showed that the high levels of polyphenols in the grape skin of wine is associated with a healthy gut microbial pattern and grape skin polyphenols could be responsible for many of health benefit associated with wine consumption [75].

Diffusible polyphenols are oxidized by PPOs to quinonic compounds. Quinones and amino acids are usually compartmentally separated in vivo as they associate reversibly and irreversibly with proteins and amino acids [89]. One junction where oxidized polyphenols meet, react and propagate their influence is in the digestive tract. Here, quinone–alpha amino acid conjugates form with nucleophiles, such as sulfhydryls, amines, amides, indoles and imidazole substituents to form imines and 1,4-Michael additions through glycation mechanisms, which if not reversed form stable adducts and aldehydes through Strecker degradation. These intermediates have germicidal activity, act as a cancer initiator and are cytotoxic or have free-radical scavenging activity [8,89]. It is intriguing to consider role for gut bacteria in redox regulation of a reversible polyphenol-quinone dynamic protecting sensitive functional groups from glycation or oxidative stress mediated damage. However, this mechanism is not well-characterized, but may add to the possibilities for wine to protect or prevent deleterious damage in the gut and elsewhere.

## 8. Wine, Mediterranean Diet and Betaine

Wine contains many anti-inflammatory molecules, such as betaine, phenolic acids, alcohol and all of which are able to lower inflammation. Folate contributes to the French paradox, since folate affects homocysteine levels and a cardio-protective role is suggested [60]. Betaine may offer another mechanism for the French paradox since it can participate in homocysteine methylation as a methyl donor and is an alternative pathway in homocysteine detoxification. Since mutations and polymorphisms in the methylene-tetra-hydro-folate reductase (MTHFR) gene and MTHFR pathway, affect its ability to process folate, leading to heart disease by increasing levels of homocysteine. However, betaine also originates from choline, which is a quaternary ammonium compound that can be oxidized irreversibly to form betaine when catalyzed stepwise by choline dehydrogenase and betaine aldehyde dehydrogenase in the liver and kidney [90]. Betaine, also known as trimethylglycine, is zwitterionic compound, which could be a potential intermediate in Wittig reactionsbyrn, especially through the Schlosser modification [91]. 

**Figure 2:**
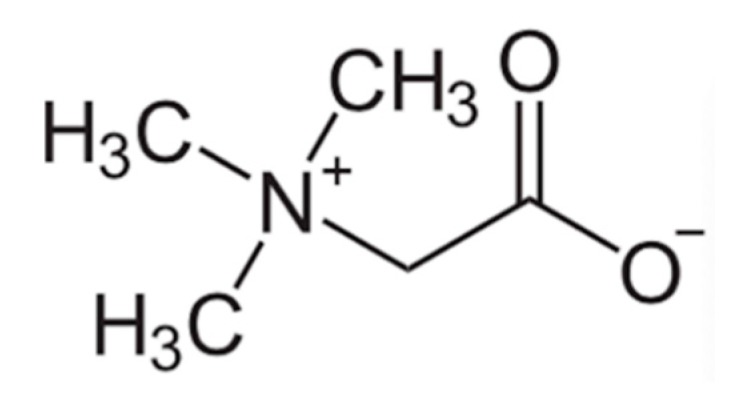
Betaine (trimethylglycine).

Betaine (See Figure 2) is another important osmolyte to stabilize tissue and cellular osmolarity, protecting against osmotic stressors like high salinity or temperature. Intracellularly, betaine aids in water retention and protects from dehydration. More importantly betaine serves as a methyl group donor in many methylation reactions and detoxification of homocysteine, a proatherosclerotic metabolite. Hyperhomocysteinemia predispose affected individuals to endothelial cell injury, to vascular inflammation and eventually to atherosclerosis. Atherosclerosis and coronary artery disease manifest when an atherosclerotic plaque blocks blood flow to coronary or other arteries. Betaine-homocysteine *S*-methyltransferase (BHMT) re-methylates homocysteine to methionine producing dimethylglycine [92]. This reaction is an alternative to vitamin B12-folate-dependent pathway for homocysteine detoxification [93].

When used as an FDA approved drug betaine is used to treat homocystinuria and liver disease but so is a combination of three vitamins folate, methylcobalamin and pyridoxine as well and offers an alternative to betaine BHMT pathway. Though it is not known whether betaine promotes cardiovascular disease through the action of select microbiota, any dichotomy that may exist between protective vs. deleterious effects is difficult to explain with current evidence. The truth may indeed be borne out from studying the microbiota of the French and co-culturing select prosaic foods with wine constituents under various incubation approaches. Human stool or fecal pools co-cultured with various compounds in a drug discovery approach should aid our understanding of microbial metabolism especially from wine constituents [94]. Uses for betaine HCl, as a dietary supplement, is attributed to many health claims, such as improving poor digestion from hypochlorhydria or achlorhydria secretion deficits. However, this seems to be a transient or temporal solution [95]. Interestingly, hyperchlorhydria can result from *Helobacter pylori* bacteria, atrophic gastritis or acid-reducing drugs, such as proton (H_2_)-receptor agonists and proton-pump inhibitors or PPIs. Moreover, *H. pylori* is implicated in heart disease [96]. 

If the explanation for the French paradox is that the French just eat more slowly than the rest of the world, then betaine proponents would argue that food breaks down in the stomach more slowly when stomach acid is inadequate. This means the inter-stomach transit time is longer due to slower stomach emptying, which could contribute to gastric reflux. Low stomach acidity has been linked to impaired absorption of vitamins B12 and C and minerals like iron, calcium and magnesium [95], all of which affect the heart and increase viral and parasitic load [97]. 

While there is no know clinical study involving co-administration of Betaine HCl with pH-dependent drugs, PPIs or certain antibiotics [98], individuals with normal gastric pH can have similar symptoms. This suggests that low gastric acid might be a symptom of another underlying problem [99]. On the other hand, some maintain that the French have less stress that the rest of the western world. While this is debatable, we can be sure that distress vs. eustress is not the optimal human condition. In that regard, chronic over-activating of the hypothalamic-pituitary-adrenal axis can be deleterious when not faced with an actual flight or fight challenge. Moreover, signs of over stressed brain include overproduction of cortisol, adrenal stress and a hypothalamic tongue. While the French are not immune from these social conditions, how they handle it is another question, perhaps for the sociologists. 

## 9. Choline, the Mediterranean Diet and Heart Disease

**Figure 3:**
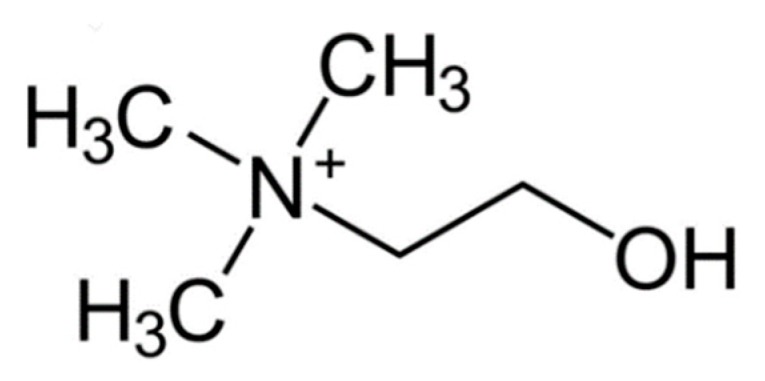
Choline.

Choline (Figure 3) is included with water soluble B complex of vitamins, which is comprised of thiamin(e), riboflavin, niacin, vitamin B6, folate, vitamin B12, pantothenic acid and biotin, is considered to be part of the eight essential vitamins, which include thiamin(e), riboflavin, niacin, vitamin B6, folate, vitamin B12, pantothenic acid, and biotin and choline [90]. These vitamins are grouped into two categories: namely, those involved in the reactions of intermediary metabolism related to energy production and redox status and those involved in the transfer of single-carbon units. Choline and the B vitamins are essential for human health [100]. 

Choline helps maintain normal brain development and is derived largely from dietary phosphatidylcholine consumption. Foods high in choline include fish, eggs and peanuts. Choline, which occur in water soluble and lipid soluble forms, and the water-soluble B vitamins help maintain proper system function and energy metabolism. Choline, as well as betaine, a choline metabolite, play important roles in reducing inflammation and for our brain and cardiovascular health. Interestingly, both of these compounds reduce plasma homocysteine levels, which a modified amino acid mainly derived from meat consumption. High levels of homocysteine are linked to increased heart disease. The author has shown a relationship between thiamin(e) and Autistic Spectrum Disorder (ASD) [101]. 

Moreover, the B vitamins thiamin(e), niacin, vitamin B6, riboflavin and pantothenic acid are required for transamination, decarboxylation, acylation, oxidation, and reduction of numerous substrates that eventually are involved in energy utilization and homeostasis. One or more of the B vitamins are important for cholesterol, steroid, amino acid, fatty acid and glucose synthesis. Several carboxylases require biotin for carbon dioxide fixation for respiration and the transmission of the energy pathway requires methylation and vitamin D, 5-MTHF (active folate), methylcobalamin (active vitamin B12), pyridoxal 5’-phosphate (active vitamin B6), betaine, choline, riboflavin 5’-phosphate (active vitamin B2) and magnesium are all needed for methyl-group transfer. Their metabolism intermingles at the pathway for conversion of homocysteine to methionine. Thiamine and pyridoxal sulfate are necessary for deoxyribonucleic acid (DNA) synthesis as is folate, which supplies single-carbon units in the pathway. There is growing evidence that some B vitamins, and choline may prevent the occurrence of developmental abnormalities and chronic degenerative or neoplastic diseases [102]. However, others suggest they may contribute to neoplastic diseases, exacerbate them or have no effect [103]. 

Heart disease is the major cause of death in the United States and for many years, physicians and researchers have been emphasizing the importance of the Mediterranean diet. Consuming a Mediterranean diet can lower the risk for heart disease and stroke. However, this diet is rich in choline and betaine and it is choline metabolism that produces betaine. Many health bloggers have argued that most adults would benefit from choline and betaine HCl supplementation but few proper studies have ever supported those claims. More evidence is needed bout the health cost vs. benefit to choline consumption but new evidence suggests that betaine and choline supplementation may even be harmful. In that regard, the cholesterol theory of heart disease and its relationship to egg consumption is not well established as causing heart disease and our Cleveland Clinic Foundation (CCF) colleagues have identified a link between eggs and phosphatidylcholine (PC) with coronary heart disease that do not involve the cholesterol theory of CHD through production of trimethylamine-N-oxide (TMANO/TMAO) [104].

## 10. Choline, Gut Microbes and the Conversion of Methylamines to TMANO/TMAO

Due to the metabolic enzymatic action of anaerobic bacteria, dietary choline, PC, betaine all produce TMANO [105]. Prior to absorption dietary choline, a nutrient found largely in eggs, organ and other animal foods, and carnitine is metabolized in the large intestine by gut microbiota to trimethylamine (TMA) and to dimethylamine and methylamine albeit in lesser amounts [106]. After absorption, host enzymes convert TMA to TMANO in the liver by liver flavin monooxygenases [107]. TMANO formation in humans is poorly understood but it is purported to enter the same metabolic pathway as its precursors, which is one of several proposed that may contribute to bacterial pathobiology of clot-formation. We have quantified TMANO levels in many rodent tissues, fluids and brain as well as in human CSF, but the origin of TMANO in the brain is not well characterized [62] but it does illustrate an aspect of the Microbiota-Gut-Brain Axis Heart-Shunt, that microbe-derived metabolites reach the brain and compartments but also have an effect on the heart.

The major precursor for TMANO in the human diet is choline, which increases urinary excretion of trimethylamine and its N-oxide [108]. Following oral intake of choline, Zhang and colleagues reported 63% of the dose as TMA and its N-oxide for D,L-carnitine (31% dose) and trimethylamine N-oxide (78% dose) [109], but failed to show ingestion of betaine, creatinine or lecithin elicited significant increases. However, in rats, creatinine feeding slightly increased urinary TMA levels [110]. TMANO elevation does not necessarily depend on gut microbiota upon consumption of dietary precursors like choline. Moreover, TMANO is eliminated rapidly in healthy adults but sustained elevation is seen in atherosclerotic patients and elevated TMANO levels are found in animal models of disease [111]. Further, TMANO is not always associated with disease or any adverse effect, and potential therapeutic applications of TMANO include protective functions in numerous organisms like bacteria where it also maintains cell volume, protecting cells from osmotic and hydrostatic damage (see concluding remarks for more putative beneficial findings).

In a study investigating TMANO formation from 46 different ingested foods, only fish and other sea-foods gave rise to significant increases in urinary trimethylamine and N-oxide [109], whereas fruits, vegetables, cereal, dairy and meats had no measurable effects on TMANO levels since they lack TMA in the first place. Serum TMANO levels are affected by not only diet and gut microbial distribution but by antibiotic administration and liver flavin monooxygenase (FMO3) activity among others. In that regard, human FMO3, which oxidizes nucleophilic heteroatom-containing xenobiotics, will also oxidize TMA in the liver to TMANO [109,112] Again, and important for this discussion, TMANO/TMAO is important to track the redox reaction mechanisms since TMANO itself can be reduced in the gastrointestinal tract to trimethylamine and dimethylamine [105,113]. In that regard, it was demonstrated that oral dosing of TMANO 78% was recovered unmodified in the urine and about half the dose was reduced to TMA in the gut, which was then re-oxidized in the liver to TMANO before excretion. So, up to half of the TMANO consumed is excreted unchanged [114]. 

Other foods have significantly more choline than eggs and a more significant impact on TMANO production. Nevertheless, this study appears to implicate meat consumption for the increased production. It appears that excessive intake of red meat in particular and foods high in choline readily increase plasma TMANO levels, which can cause blood clots and increases the risk of heart disease [115]. For example, a study on dietary effects of 46 different foods on urinary excretion of TMANO in 6 human volunteers showed eggs had largely no effect on TMANO excretion, but many sea foods did as seafood contains TMA and the TMANO is derived from TMA via direct oxidation of the amino group. In addition, fish contain lower amounts of choline than eggs, which may argue for the direct oxidation and stochastic conversion mechanism of TMA to TMANO over enzymatic metabolism or at least offer an additional pathway.

One approach to preventing heart disease from this research group was to inhibit host enzymes that convert TMA to TMANO, but this caused the formation of pro-atherogenic intermediates [115] and the approach presumably caused liver damage and accumulation of toxic TMA. The better approach may be to target specific gut microbes to prevent TMA formation in the first place. Ongoing efforts to inhibit TMANO production without understanding why in this study vegetarians have lower amounts of TMANO and choline, and even took choline supplements, than those who ate meat confounds any proposed hypotheses and question the use inhibitors at this point. Although there are cultural differences, dietary preferences, and prebiotics which could offer explanations for some of these effects, there are still genetic and epistatic and environmental unknowns to be elucidated. For example, it is intriguing that Indian, French and Mediterranean people, whom all eat high-choline and high betaine-containing foods, or are largely vegetarian, have relatively good heart health in spite of likely having the same intestinal bacterial microbiota. 

When choline is supplemented orally over two months, production of trimethylamine N-oxide increased ten-fold [105] The CCF researchers found that the compounds present in animal products increase the levels of TMANO, which may directly cause platelets to form blood clots. This finding helped link chronic diseases, such as obesity, atherosclerosis and diabetes with the gut microbiota. What it does seem to indicate is a strong microbial involvement in pathogenesis and CHD risk. However, these findings and newer ones on microbial enzyme transformation add considerable confusion to the story of the French paradox as PC, choline and betaine are compounds that share routes of metabolism and production. While this study involved both a broad spectrum antibiotic and animal studies using germ-free mice and suggests heart disease has a bacteria-derived biochemical component to its pathogenesis, it leaves many questions unanswered. TMANO may have a role in protecting the brain and certainly the case has been made for PC and choline in brain health.

However, flavonoids and lignans form wine have both antiplatelet and antithrombogenic activity, which is mediated through binding of cyclooxygenase [116]. Their anti-thrombogenic activity and anti-inflammatory properties are mediate through inhibition of the metabolism of arachidonic acid [116], which is an important inflammatory precipitating molecule by neutrophils that use lipoxygenase and arachidonic acid for chemotaxis. Nevertheless, these initial studies lack full exploration of not only the consortium of bacteria involved, but they create more questions than answers about consuming eggs and meat vs. vegetarianism and beg for much more basic science. Given the confusion around choline, betaine and since Dietary Reference Intakes (DRIs) are largely determined through human observational and depletion-repletion studies. However, it is difficult to ascertain a dietary recommended daily allowance (RDA) for choline intake, which although was established, adequate intake is expected to be 425 milligrams per day for women and 550 milligrams a day for men. [117]. Nevertheless, if choline-containing foods really are key to TMANO production *in vivo*, and if high TMANO causes heart disease, then one would expect greater rates of CHD among people who eat more fish, whole grains and choline rich foods, since these are a fair component of a Mediterranean diet and these foods produce more TMANO. Yet, this is the opposite of what cohort studies indicate, as eating more fish especially wild caught cold-water, fatty fish like salmon, rich in omega 3 fatty acids has consistently been shown in randomized controlled trials to reduce the risk of death from coronary heart disease, mortality and sudden death [118]. Recently, blood levels of long-chain omeg-3 fatty acids were found to be strongly associated with a reduced risk of sudden death among men without prior cardiovascular disease [119] and heart rate is positively associated with risk of sudden death in men [120].

## 11. TMANO-mediated Damage or Health Benefits

TMANO is suspected of being many things from pharmacological therapeutic agent to evolutionary remnant of an osmolytes-regulation system among others. Among others, mutant protein chaperone [121] and helpful against diverse ocular diseases, such as cataracts [122], glaucoma, corneal dystrophies and other ocular diseases [123]. Moreover, several mechanisms affect TMANO homeostasis in vivo and plasma levels are dependent upon numerous factors including, diet, microbial composition of the gut and medications other than antibiotics. However, anything that controls the rate of TMA formation or its excretion, like the permeability to TMA and TMANO of the blood intestinal barrier or the excretion of the methylamines [113,124] will also affect TMANO levels. Further, kidneys maintain TMANO homeostasis by regulating water-electrolyte balance and excretion rates are also regulated by functioning healthy kidneys. 

In regard to kidney function and renal insufficiency, TMANO’s effect on increased atherosclerosis and heart disease risk in humans and rats may involve its interaction with angiotensin II [125] which, interestingly, affects cholesterol metabolism [126]. Moreover, related to kidney function, angiotensin converting enzyme inhibitors (ACE-I) are used to treat cardiovascular, kidney and heart diseases and are known to reduce cardiovascular mortality [127]. When the ACE-I Enalapril was used in patients and rodent models, it reduced plasma TMANO level, but it is known to also affect composition of gut bacteria and the plasma level of several gut bacteria-derived metabolites including the generic bacterial maker indoxyl (or indican) [123] The mechanism for the enalapril reduction of plasma TMANO is not well characterized and an increased urinary excretion of TMANO or methylamines can’t be ruled out. However, over time enalapril may disrupt kidney function, disrupt electrolyte levels [128] and lead to kidney failure. When the same group studied enalapril-treated rats, no markers of kidney failure were found [129] with the bacterial marker indican (Indoxyl sulfate) were observed. It is important to note that Enalapril is a diuretic and natriuretic and rats given this drug had higher water intake that controls [130] and effect of ACE-I on the gut–blood barrier permeability and methylamine homeostasis is not known [123] but this affect could have benefit in cardiovascular disease that involve urinary excretion of deleterious metabolite. 

## 12. TMANO and Leaky Gut

In regard to leaky gut, leaky brain and other barrier permeability states, it is suggested that TMANO might cause BBB or other barrier disruption by reducing the expression of tight junction protein-1 ZO-1 and claudin-5 [131]. Moreover, leaky gut is a serious consideration for the transmission of bacteria and material components or Lipopolysaccharide (LPS) into the systemic circulation. Further, leaky gut and gluten sensitivities have been considered to cause or contribute to inflammatory states especially with the autoimmune disease Celiac, which is characterized by inflammation of the intestinal mucosa in the small intestine due to an immune response and the loss of tolerance to dietary gluten. Moreover food sensitivity and emerging food glycotoxins that have been linked to systemic inflammation, especially in regard to celiac disease (CD), depression and psychiatric comorbidities [3]. 

Inflammation in the body may lead to direct and even disruptive effects on the brain via the blood–brain barrier and blood-gut barrier. This may be perhaps why we find TMANO in the brain. During systemic inflammation, whether from infection or LPS translocation, the BBB may undergo changes in patients with neurological disease to become abnormally sensitive to the effects of systemic inflammation [6]. Conversely, several lines of evidence suggest that TMANO may play a protective role in cardiovascular system by reducing deleterious effects of oxidative stress. For example, it has been found that oral supplementation of L-carnitine increases plasma TMA and TMANO levels and decreases markers of oxidative stress and vascular injury, such as vascular cell adhesion molecule-1, soluble forms of intracellular adhesion molecule-1 (ICAM 1), and malondialdehyde [132]. Furthermore, treatment with TMANO has been found to inhibit the negative effects of oxidative stress in streptozotocin diabetic rats [133] and human neuroblastoma cells [134]. However, the mechanisms are not clear. TMANO has also been found to act as an electron acceptor in bacteria [135]. Therefore, it may neutralize electrons that leaked from the mitochondrial electron transport chain reducing the formation of reactive oxygen species.

## 13. Pathogenic Bacteria and Disease 

In addition to heart disease, Alzheimer disease, schizophrenia, ASD and autism are increasingly expected to be influenced by the gut microbiota. In that regard, it is well established that bacteria can directly cause human blood and plasma to clot. Moreover, these coagulation states can be acquired from systemic diseases or sequalae associated with an increased risk of thrombosis. For instance, hyperlipidemia, malignancy, myeloproliferative disorders, use of oral contraceptives, pregnancy, and even diabetes all are known to cause hyper-coagulation [136]. Previous conventional thought suggested this process was lost during vertebrate evolution. One key to clot formation is the location of the bacteria or infection site but coagulation only occurs when bacteria cluster into a foci of critical mass [137]. Since we know choline does feed select gut bacteria and it is known certain bacteria can produce clot-forming compounds and contribute to a hyper-coagulability or secondary hypercoagulable states in humans, perhaps focusing on the bacteria, fungi and parasites would render a fuller understanding of these diseases and advance medicine in ways never before imagined. While this is important for possibly increasing stroke or heart attack risk, it begs the question as to whether or not innocuous and even beneficial foods can be antagonistically pleiotropic with respect to the action of select gut bacteria. 

It is important to revisit redox mechanisms in regard to TMANO as it potentially affects oxidation-reduction mechanisms, impair fatty acid oxidation in cardiac mitochondria [138] and serve as an electron acceptor in animal intestines for anaerobic metabolism of various bacteria like *Enterobacteriaceae* [135]. These researchers found TMANO to support oxidative phosphorylation in *Enterobacteriaceae*, which is a common bacteria in mammals. In the Colon, anaerobic respiration with TMANO was at the same time able to inhibit the facultative pathogen *Staphylococcus aureus* and other bacterial growth [139]. Facultative anaerobes, such as bacteria belonging to the groups *Bacillus, Staphylococcus, Corynebacterium, Fusobacterium* and many members of the phylum *Proteobacteria* are opportunistic species, especially *Enterobacteriaceae*, thrive in an inflamed environment [140]. Substantial evidence exists that TMANO may play an important role in the global carbon and nitrogen cycles [141] and we should mention that oral infections and bacteria play a role in heart disease.

Further, carnitine, abundant in meat and high energy drinks may responsible for increasing production of smaller amounts of TMANO from bacterial metabolism. What we don’t know is which specific bacteria, are involved, although *Prevotella* bacteria is known to produce higher levels of TMANO. Intestinal microbiota metabolism of L-carnitine, which promote atherosclerosis [126], is found from whole grain and not animal product consumption that is associated with higher levels of *Prevotella* bacteria [142]. The author speculates that meat and carnitine-loving bacteria are key players. Alternatively, *Clostridia difficlie* may be another culprit and find that *Clostridia difficlie* is lower in vegetarians vs. carnivores (unpublished observations). Yet, carnitine supplementation coupled with alpha lipoic acid, is able to protect the heart and brain from oxidative stress, aging and possibly bacterial action and may be one solution to the problem. Moreover, polyphenols help prevent inflammation and oxidation and thus help prevent downstream effects from these processes, which are implicated in almost every disease [7].

So, while it cannot be concluded that choline, betaine and carnitine are responsible for strokes or heart disease, we should not eliminate them from the diet. The Cleveland Clinic Foundation researchers demonstrated that a Mediterranean diet altered the activity of gut microbes, which may be the very node that modifies bacterial risk in humans. Nevertheless, scientists should explore bacteria-host co-metabolism with phenolic acids, antioxidants and polyphenol administration and sequence the microbiota of the heart healthy for genetic associations or polymorphisms to gain a better understanding. Regardless, it may be best to consume TMANO-promoting foods in moderation, or focus on eating more vegetation or drink a glass of red wine per day and so on or at least until all the facts are made clear. Other foods in the aforementioned dietary challenge like fish, whole grain etc. may increase Prevotella colonization [143].

Polyphenols help lower systemic inflammation, improve metabolic syndrome and favorably affect the microbiota as a prebiotic. They also induce the microbiota to release prebiotics, such as SCFAs [144]. In addition to using prebiotics to modulate the microbiota, a useful approach to inhibit or counter damaging metabolic products from harmful bacteria may be to use beneficial probiotics to change the kinetics of the harmful bacterial species or compete for substrates. For instance, there are many rare and valuable enzymes useful for chemical synthesis. However, physiologic constraints like instability, unfavorable thermodynamics or poor kinetics do severely limit their usefulness in particular applications. Harnessing the microbiota in fermenters, or other synthetic approaches, to synthesize medicinal compounds or natural products as drugs or antibiotics could help deliver these new treatments quickly and on a large scale. For example, a research group from Tufts recently employed a microbial approach to surpassing isomerization catalysis barriers for D-Tagatose biosynthesis [145]. The value of this rare ketohexose sugar as a commodity for its safety, per United States Department of Agriculture, which is (generally regarded as safe or given GRAS designation) as a substitute for diabetic use and it has a low glycemic index, similar sweetness to sucrose and a low calorie content [145]. The same authors created an enzymatic reaction system using Lactobacillus *sakei* and Lactobacillus arabinose isomerase to isomerize of D-galactose to D-tagatose. They overcame low catalytic efficiency and low thermal stability by encapsulating the enzyme in a gram-positive Lactobacillus *plantarum*, which was chemically permeable and enabled high reaction rates at high temperatures [145]. What this approach serves to illustrate is proof of concept that the application of microbiology to inhibit bacterial production of deleterious substrates like Trimethylamine or downstream products like TMANO.

## 14. Conclusions

Since polyphenols and antioxidants are able to counteract many deleterious bacteria-driven mechanisms, it follows that unwanted co-metabolism could be inhibited by prosaic foods rather than with inhibitors or drugs. Natural compounds are preferred over synthetic ones since they are readily available, inexpensive, and comparatively less toxic. However, they are very difficult to synthesize commercially. Nevertheless, support for this notion comes from the Cleveland Clinic Foundation researchers, themselves, who found that prosaic foods like cold-pressed extra virgin olive oil, balsamic vinegar and grape seed oils could also inhibit microbes from transforming meat, choline, betaine and carnitine into metabolic by-products of TMANO in animal studies, but they did not explore antioxidants or redox mechanisms directly. 

There is considerable conjecture about vitamin supplementation and choline is not exempt. High levels of TMANO are associated with a greater risk of heart attack and stroke. However, according to a large Finnish study, adequate intake of choline reduced the risk of dementia [146], cardiovascular disease and cancer. The data for the study were derived from the Kuopio Ischemic Heart Disease Risk Factor Study, which analyzed approximately 2500 men aged between 42 and 60 for their dietary general health and lifestyle habits. They observed that dietary phosphatidylcholine intake (largely from eggs and meat) was associated with reduced dementia risk. Phosphatidylcholine intake was also associated with enhanced cognitive performance. This is not surprising, given that choline is necessary for the formation of acetylcholine a key neurotransmitter. If one goes further to consider the microbiota-gut-brain axis as both culprit and cure to not only prevent atherosclerosis, but prevent brain and other disorders as well and do it through microbiota-host co-metabolism [147]. Key to this hypothesis is both diet and targeting select gut bacteria to either prevent or treat disease. In other words, using “Bugs as Drugs” or using antimicrobial substances like polyphenols to modulate health and disease. So, while possible to prevent the diet-induced heart disease that starts from the gut, researchers still don’t understand all this would entail. But it is clear that funding basic science is still a valuable investment and there should be so much more of it. 

Technical aspects typical with human studies like the TMANO studies make our determination difficult such as those from confounding factors like diet and the constitution of the study subjects. For example, kidney function is important in toxin clearance and we see filtration rate and kidney function from some study participants but any impaired urinary clearance of TMANO or impaired kidney function would diminish the results. The conjecture is supported in the New England Journal of Medicine paper, where the highest levels of TMANO had an average glomerular filtration rate (GFR) of 69 mL/min, which is indicative of a reduced filtration determined as a GFR between 60–89 mL/min. In their defense, however, a healthy volunteer study was conducted where the results appeared to hold. Further, the microbial milieu should be determined throughout these studies as some gut microbiota, i.e., Prevotella species are known to predispose one to increases in TMANO production [148].

**Figure 4:**
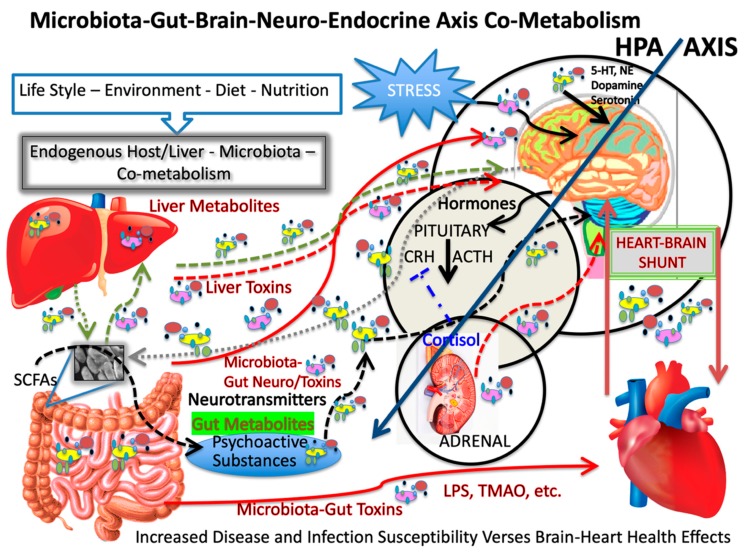
Overview of the Microbiota-Gut-Brain Axis-Heart Shunt with emphasis on stress, and the French paradox, where gut-derived microbial toxins and liver-derived toxin transformation contribute to heart disease and dysbiosis to mental disease. Whereas the same systems when functioning optimally produce beneficial SCFAs, neurotransmitters and hormones. The bidirectional pathway for toxin and psychoactive substance production affect both the brain and the heart. Conversely, gut derived hormones, peptides, small molecules and neurotransmitters have a useful role in health and in both prevention of heart and brain diseases.

In summation, we cannot say that choline and betaine are protective or deleterious as food consumption goes, but they do affect many aspects of our health, especially when considering potential transformation by enteric bacteria in the colon. Nevertheless, consuming excess choline-rich foods or when supplements are added, may increase TMANO levels and could increase the risk of heart disease. Yet, the betaine, when consumed in red wine, however, may be protective and red wine with a choline-rich meal may impart similar protection from choline formation via a microbial route. As yet, no one has established a clear mechanism for how the French enjoy their food or any heart disease-sparing mechanism and more work is necessary to address these aspects, which we elucidate here both for the microbiota gut brain heart shunt and in neurodegenerative disease. This work calls for reexamining many experimental approaches and couple them with exploring the microbiota of the French. Taken together, we argue that oxidative stress, through microbial action and metabolism, could contribute to TMANO formation but other factors may be more important in modulating heart and cerebrovascular disease and related forms of AD, VaD and dementia (See Figure 4 for an overview). While the figure is somewhat complicated, it is clear that the answers to the questions surrounding the French paradox are complicated as well. A quote paraphrased from “Sleeper” (1973, United Artists), a futuristic Woody Allen comedy, in which this character Miles says ”I used to run a health food store, The Happy Carrot in the West Village one day I went in for routine surgery only to wake up two hundred years later to find everything that I was told was good for me is bad for me and everything that was bad for me is good for me.” It often seems like this when exploring the paradoxical, which means things do not always make sense and can be contrary to the expected. Nevertheless, we have much more to learn about this important topic and the roles the microbiota gut brain axis play in heart health, heart disease and neurodegenerative diseases.

## Abbreviations

microbiota-gut-brain	MGB
trimethylamine-*N*-oxide	TMANO/TMAO
Autistic Spectrum Disorder	ASD
tumor necrosis factor	TNF
endothelin-1	ET-1
reactive oxygen species	ROS
reactive nitrogen species	RNS
lipopolysaccharide	LPS
Peroxynitrite	ONOO
nitric oxide	NO
low-density lipoprotein	LDL
celiac disease	CD
vascular dementia	VaD
hydrogen sulfide gas	H_2_S
central nervous system	CNS
hydrogen sulfide	H_2_S
methylene-tetra-hydro-folate reductase	MTHFR
G-protein coupled receptor kinase	GRK
short chain fatty acids	SCFA
Dietary Reference Intakes	DRI
recommended daily allowance	RDA
polyphenol oxidase	PPO
Phosphatidylcholine	PC
Betaine-homocysteine *S*-methyltransferase	BHMT
Trimethylamine	TMA
glomerular filtration rate	GFR
angiotensin converting enzyme inhibitors	ACE-I
